# Do Cancer Patients Tweet? Examining the Twitter Use of Cancer Patients in Japan

**DOI:** 10.2196/jmir.3298

**Published:** 2014-05-27

**Authors:** Atsushi Tsuya, Yuya Sugawara, Atsushi Tanaka, Hiroto Narimatsu

**Affiliations:** ^1^Yamagata University Graduate School of Science and EngineeringYamagata UniversityYonezawaJapan; ^2^Medical Informatics focus, Environmental Life Science majorYamagata University Graduate School of Medical ScienceYamagata UniversityYamagataJapan; ^3^Department of Public HealthYamagata University Graduate School of MedicineYamagata UniversityYamagataJapan

**Keywords:** communication, co-occurrence, Internet, leukemia, Web 2.0

## Abstract

**Background:**

Twitter is an interactive, real-time media that could prove useful in health care. Tweets from cancer patients could offer insight into the needs of cancer patients.

**Objective:**

The objective of this study was to understand cancer patients’ social media usage and gain insight into patient needs.

**Methods:**

A search was conducted of every publicly available user profile on Twitter in Japan for references to the following: breast cancer, leukemia, colon cancer, rectal cancer, colorectal cancer, uterine cancer, cervical cancer, stomach cancer, lung cancer, and ovarian cancer. We then used an application programming interface and a data mining method to conduct a detailed analysis of the tweets from cancer patients.

**Results:**

Twitter user profiles included references to breast cancer (n=313), leukemia (n=158), uterine or cervical cancer (n=134), lung cancer (n=87), colon cancer (n=64), and stomach cancer (n=44). A co-occurrence network is seen for all of these cancers, and each cancer has a unique network conformation. Keywords included words about diagnosis, symptoms, and treatments for almost all cancers. Words related to social activities were extracted for breast cancer. Words related to vaccination and support from public insurance were extracted for uterine or cervical cancer.

**Conclusions:**

This study demonstrates that cancer patients share information about their underlying disease, including diagnosis, symptoms, and treatments, via Twitter. This information could prove useful to health care providers.

## Introduction

### Twitter: A Novel Social Media

Twitter is a free social networking and micro-blogging service that enables its millions of users to send and read each other’s “tweets”, or short messages limited to 140 characters. The users themselves determine whether their tweets can be read by the general public or should be restricted to preselected “followers”. As of March 2012, the service had more than 200 million registered users and processed about 400 million tweets per day [1,2].

A recent analysis of the “Twitter stream” revealed that a substantial proportion of tweets contain general chatter, that is, user-to-user conversations that are of interest only to the parties involved, links to interesting pieces of news, or spam and self-promotion [1]. Despite the high level of noise, the Twitter stream does contain useful information. Recently, we and other researchers demonstrated that Twitter is emerging as an important channel for communicating about cancer [3-7]. Many recent news events or scientific issues have been documented and discussed via Twitter directly from users on the site in real time [8]. Although the information that one tweet includes is limited, Twitter can convey more immediacy with interactivity than website homepages or blogs [1,9-12], such as the Association of Cancer Online Resources [9]. Thus, Twitter has the potential to play a different role in sharing medical information among patients.

### Twitter in Cancer Patients

In a recent case study, we demonstrated that Twitter networks of cancer patients centered on active users and that these networks could provide psychological support for cancer patients [4]. Because of certain restrictions of the search tool, the study was not able to conduct a large-scale comprehensive qualitative analysis. Therefore, in the present study, we examine cancer patients’ social media usage by analyzing the data with a text mining method using an application programming interface (API) [2]. Thus, we were able to comprehensively analyze the Twitter data of cancer patients on a large scale.

## Methods

### Search for Twitter Accounts of Cancer Patients

A search was conducted of every publicly available user profile on Twitter in Japan. We examined the number of user accounts in which the names of cancers are described in the profile. The search terms included breast cancer, leukemia, colon cancer, rectal cancer, colorectal cancer, uterine cancer, cervical cancer, stomach cancer, lung cancer, and ovarian cancer. These names were alternatively searched using “cancer” in the Japanese hiragana and katakana writing system and in Chinese characters. The site used for the profile search was “16 (one-six) Profile Search β Version for Twitter” [13], which enabled us to search, in addition to profiles, the number of follows, followers, tweets, lists, registered dates, and last posted dates. The search was conducted on August 18, 2013. This study was approved by the Institutional Review Board at Yamagata University Faculty of Medicine (H24-133).

### Content Analysis of Tweets

Using Twitter API, the latest tweets (maximum 200 tweets) from each account, found after the above search, were gathered. Twitter API is a function officially provided by the organization that operates Twitter to Twitter application developers in order to provide useful and convenient functions to Twitter users. By incorporating Twitter API into an application, the application developer can add Twitter functions such as Twitter search results or obtaining tweets from Twitter accounts [14].

First, tweets obtained from each account through Twitter API were separated onto different lines with a period “.”. Subsequently, these were broken down into morphemes (“words”) using the Japanese language morpheme analysis software ChaSen (from the Nara Institute of Science and Technology, Japan). Here, the words were represented in their original forms. Nouns were then extracted from these words and were listed on separate lines. These nouns (“noun group”) listed in separate lines were then grouped together by account. Occasionally, verbs and adjectives are also extracted with text mining. However, in the present study, we did not extract verbs and adjectives for the following reasons: (1) difficulties in dealing with negative sentences, and (2) low percentage of the part of speech of the extracted word. In addition, nouns obtained that were synonyms were integrated into one noun. Synonyms were determined by the authors by referring to WordNet Web search services [15]. Dictionaries that contained words obtained from the descriptions on websites were used as the default for ChaSen (“cancer information services” [16] and “good health care” [17]).

Tweets were obtained during the following dates and times: 0:39–2:52 on August 19, 2012, for stomach cancer, colon and colorectal cancer, and leukemia tweets; 14:40–17:24 on August 20, 2012, for uterine cancer, breast cancer, and lung cancer tweets.

### Generation of Co-Occurrence Networks

**Figure 1:**
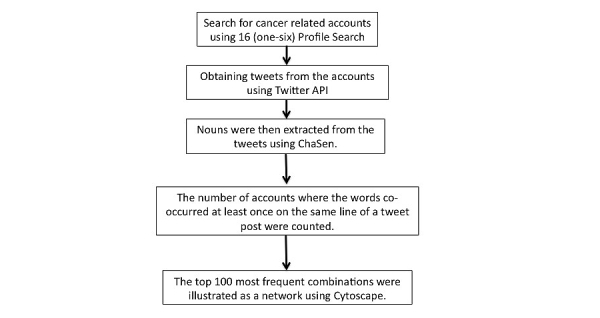
Procedure for generating the co-occurrence network.

The procedure of generating the co-occurrence network is shown in Figure 1. Co-occurrence is the relation between the keywords that appear together in each tweet; thus, co-occurrence means a close relationship between words. In this study, we demonstrate the features of tweets by cancer patients by analyzing the co-occurrence of keywords.

To accomplish this, we created co-occurrence networks using the following procedure: (1) the tweets from the cancer-related accounts were broken down into words using ChaSen, (2) from the noun groups that were combinations of two words, we counted the number of accounts where the words co-occurred at least once on the same line of a tweet, and (3) from the word combinations that co-occurred on the same line of a tweet, the top 100 most frequent combinations (the top 100 in number of accounts) were illustrated as a network with words depicted as nodes and combinations as links. Network analysis software Cytoscape [18] was used for the illustration. We first used the spring model as a node placement rule and subsequently made adjustments such that each word and each link overlapped as little as possible. The spring model is a method that can illustrate networks from the perspective of evenness of side length as well as uniformity and symmetry of node distribution. It regards each side as a spring that follows Hooke’s law and each node as an electrically charged particle that follows Coulomb’s law, and the layout is established by determining the equilibrium state [19].

In the method we used to create co-occurrence networks in this study, as a way to handle the high frequency of extremely specialized tweets, the co-occurrence frequency of co-occurrence networks was defined as the number of accounts where words co-occurred in tweets, rather than the number of co-occurrences of words, which is typically done when creating co-occurrence networks. This then prevented extremely specialized words completely unrelated to cancer from appearing in the co-occurrence networks.

## Results

**Figure 2:**
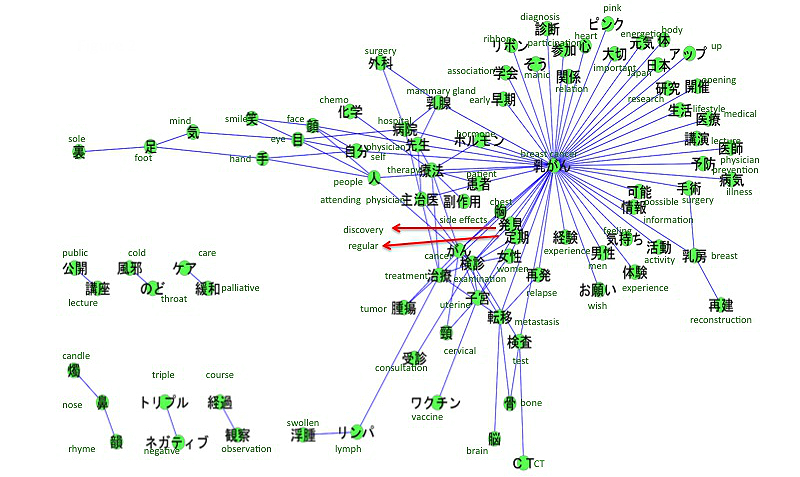
Co-occurrence network of cancers: breast cancer.

**Figure 3:**
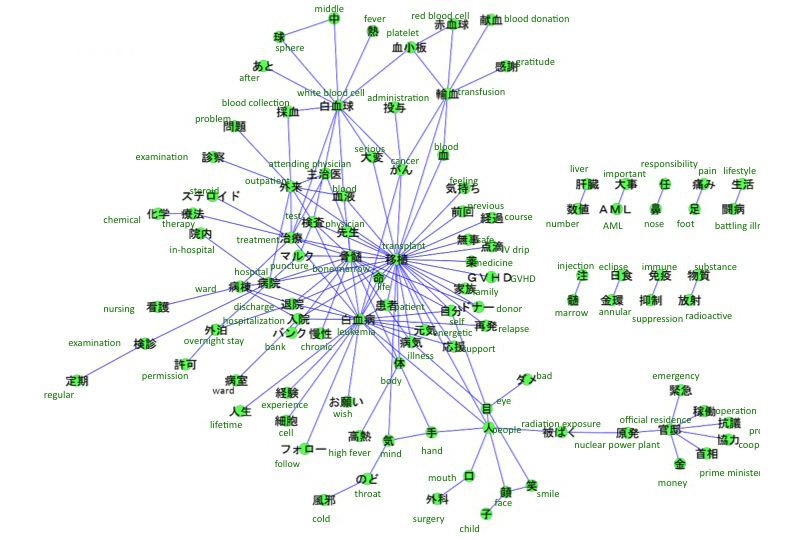
Co-occurrence network of cancers: leukemia.

**Figure 4:**
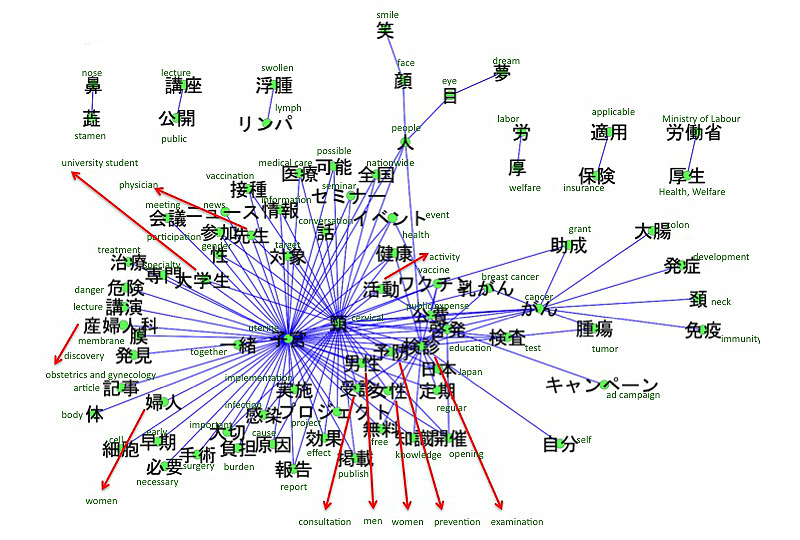
Co-occurrence network of cancers: uterine and cervical cancer.

**Figure 5:**
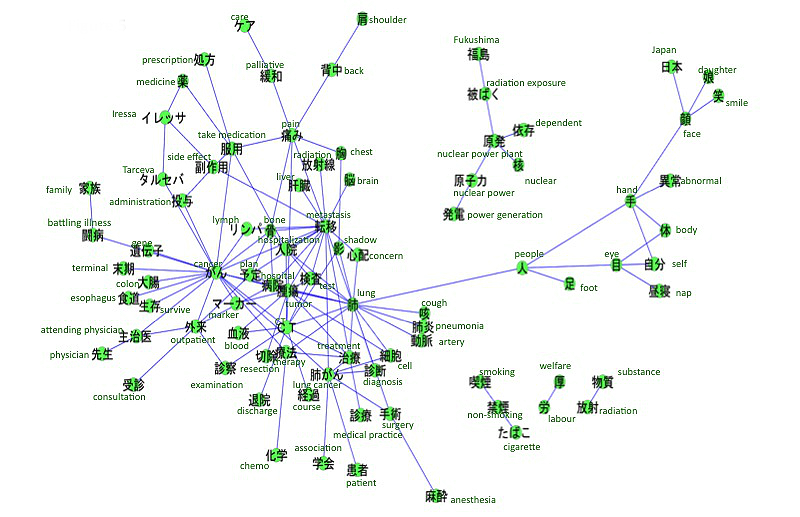
Co-occurrence network of cancers: lung cancer.

**Figure 6:**
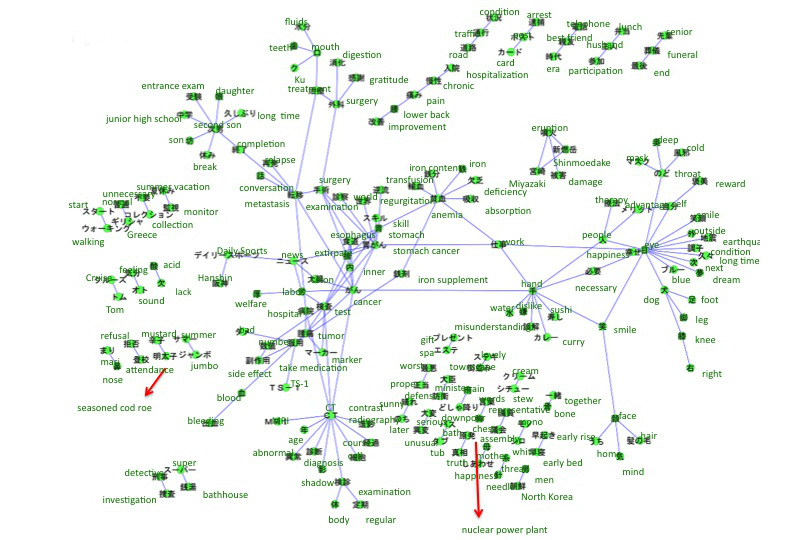
Co-occurrence network of cancers: stomach cancer.

**Figure 7:**
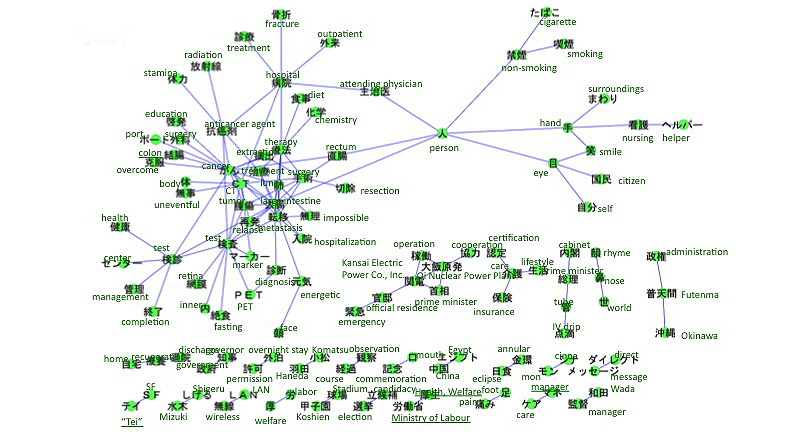
Co-occurrence network of cancers: colon and colorectal cancer.

The accounts we searched included references to breast cancer (n=313), leukemia (n=158), uterine and cervical cancer (n=134), lung cancer (n=87), colon cancer (n=64), and stomach cancer (n=44). The co-occurrence networks of those cancers are shown in Figure 2-7. Table 1 summarizes the keywords from tweets related to different types of cancer. Each cancer has a unique network conformation. The keywords included words about diagnosis, symptoms, and treatments for almost all cancers.

**Table 1:** Summary of keywords in tweets according to cancer type.

	Diagnosis	Symptoms	Treatments	Others
Stomach cancer	CT^a^, MRI^b^, tumor marker	Lumbago, TS-1, side effects	Anti-cancer drug, TS-1, administration of iron	Not available
Colon and colorectal cancer	CT, PET^c^	ELPLAT, side effects	Chemotherapy, diet	Nursing care
Cancer of uterus and cervical cancer	Not available	Lymphedema	Not available	Educational activity, screening, not covered by health insurance, vaccination, official support
Lung cancer	CT	Metastasis, shoulder pain, back pain, Iressa, side effects	Anti-cancer drug, Iressa, Tarceva	Palliative care
Breast cancer	Self-diagnosis	Metastasis, lymphedema	Chemotherapy, hormonal treatment	Palliative care, the pink ribbon
Leukemia	Liver function test	Liver function test, foot pain, immunosuppression, GVHD^d^	Chemotherapy, steroid treatment, transfusion of red blood cells, platelet transfusion	AML^e^, hematopoietic stem cell transplantation

^a^CT: computed tomography.

^b^MRI: magnetic resonance imaging.

^c^PET: positron emission tomography.

^d^GVHD: graft-versus-host disease.

^e^AML: acute myeloid leukemia.

## Discussion

### Comprehensive Analysis of Tweets

In this study, we used an information technology procedure to comprehensively analyze the content of cancer patients’ tweets. In previous studies, researchers verified each individual tweet, but this method restricted the range of Twitter information that could be obtained [4]. Moreover, a notable point of this analysis method was that we were able to exclude tweets unrelated to the diseases of interest. Using our method, information on tweets related to specific diseases can now be collected efficiently. Although we used this method to evaluate tweets from cancer patients, in the future, we plan to apply this method to the study of other diseases, for example, lifestyle-related diseases.

Twitter data can be obtained from a variety of sources. In this study, we used Twitter API because it uses an automated approach to data retrieval and is free of charge. However, the number of tweets retrieved through Twitter API is capped at approximately 1% of all tweets, with no assurance of a random or representative sample [2]. Thus, retrieving Twitter’s full data stream through automated dashboard vendors or a Twitter data reseller may provide further findings.

### Tweets Related to the Cancers

This study found that information related to cancer, such as treatment, diagnosis, and symptoms, is shared among cancer patients on Twitter (Table 1). Furthermore, the extracted keywords were considered to be medically important for that specific disease, reflecting the fact that cancer patients use Twitter as a tool for sharing medical information. Additionally, depending on the type of cancer, it was clear that there were specific characteristics to the tweet content. For example, in uterine or cervical cancer and breast cancer, there were keywords not related to immediate medical care, for example, “cervical cancer vaccine” for uterine or cervical cancer and “pink ribbon” for breast cancer. These most likely indicate that patients are also affected by the heightened social interest in a cervical cancer vaccine [20] and the social excitement of the pink ribbon movement. These topics were also covered by regular news media, such as TV or newspaper. This indicates that the content of tweets can be affected by those media.

### Conclusions and Future Directions

We indicated in a previous study [4] that Twitter is useful for cancer patients to exchange ordinary information. As industries obtain and utilize tweet information from Twitter as marketing tools, health care will be able to retrieve, study, and make use of tweet information. In this study, we comprehensively and efficiently collected tweet information related to diseases, demonstrating that information about cancer patients can be collected on social media. Effective use of this information will be helpful in developing cancer care that better suits the patients’ needs. For example, health care providers can more effectively give information or medical services to patients, resulting in an increase in patient satisfaction.

## Abbreviations

API: application programming interface
